# Artificial Intelligence Applications in Ophthalmology

**DOI:** 10.31662/jmaj.2024-0139

**Published:** 2024-09-13

**Authors:** Tetsuro Oshika

**Affiliations:** 1 Department of Ophthalmology, Faculty of Medicine, University of Tsukuba, Tsukuba, Japan

**Keywords:** Artificial intelligence, Deep learning, Retinal diseases, Anterior segment diseases, Smartphone

## Abstract

Ophthalmology is well suited for the integration of artificial intelligence (AI) owing to its reliance on various imaging modalities, such as anterior segment photography, fundus photography, and optical coherence tomography (OCT), which generate large volumes of high-resolution digital images. These images provide rich datasets for training AI algorithms, which enables precise diagnosis and monitoring of various ocular conditions. Retinal disease management heavily relies on image recognition. Limited access to ophthalmologists in underdeveloped areas and high image volumes in developed countries make AI a promising, cost-effective solution for screening and diagnosis. In corneal diseases, differential diagnosis is critical yet challenging because of the wide range of potential etiologies. AI and diagnostic technologies offer promise for improving the accuracy and speed of these diagnoses, including the differentiation between infectious and noninfectious conditions. Smartphone imaging coupled with AI technology can advance the diagnosis of anterior segment diseases, democratizing access to eye care and providing rapid and reliable diagnostic results. Other potential areas for AI applications include cataract and vitreous surgeries as well as the use of generative AI in training ophthalmologists. While AI offers substantial benefits, challenges remain, including the need for high-quality images, accurate manual annotations, patient heterogeneity considerations, and the “black-box phenomenon”. Addressing these issues is crucial for enhancing the effectiveness of AI and ensuring its successful integration into clinical practice. AI is poised to transform ophthalmology by increasing diagnostic accuracy, optimizing treatment strategies, and improving patient care, particularly in high-risk or underserved populations.

Ophthalmology has high affinity for artificial intelligence (AI) because of several intrinsic factors. This field heavily relies on various imaging modalities, such as anterior segment photography, fundus photography, optical coherence tomography (OCT), fluorescein angiography, OCT angiography, automated perimetry, corneal topography, and anterior segment OCT, which generate large volumes of high-resolution digital images. These images provide rich datasets for training AI algorithms, enabling precise diagnosis and monitoring of various ocular conditions. Continuous advancements in AI technology, coupled with collaborative research and open data initiatives, further enhance the development and adoption of AI in ophthalmology ^(1), (2)^. These factors create a fertile ground for AI integration, promising significant improvements in diagnosis, treatment, and patient care. In this article, the author reviews the progress and current trends of AI applications in ophthalmology.

## 1. Differential Diagnosis of Retinal Diseases

The identification and management of retinal diseases largely depend on image recognition. However, the distribution of ophthalmologists varies across countries and regions, limiting access to regular screening for people in underdeveloped areas. Even in developed countries, experienced ophthalmologists need to screen a huge volume of retinal images, which can consume substantial amounts of their time, detracting from other critical tasks.

**Figure 1.**
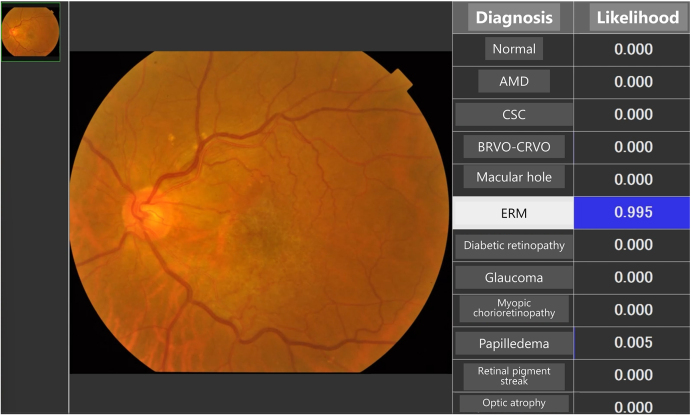
AI application for the differential diagnosis of 11 retinal diseases. This system was generated based on the big data stored in the Japan Ocular Imaging Registry ^(6)^.

These manpower and time-consuming issues have led to a growing interest in the establishment of reliable and cost-effective methods for screening retinal diseases. The capability of AI to differentiate various retinal diseases substantially enhances diagnostic accuracy and treatment planning ^(3), (4), (5), (6)^. AI algorithms can distinguish between different types of retinal diseases with high precision (Figure 1), which is crucial for determining appropriate treatment protocols and monitoring disease progression.

## 2. Differential Diagnosis of Corneal Diseases

**Figure 2.**
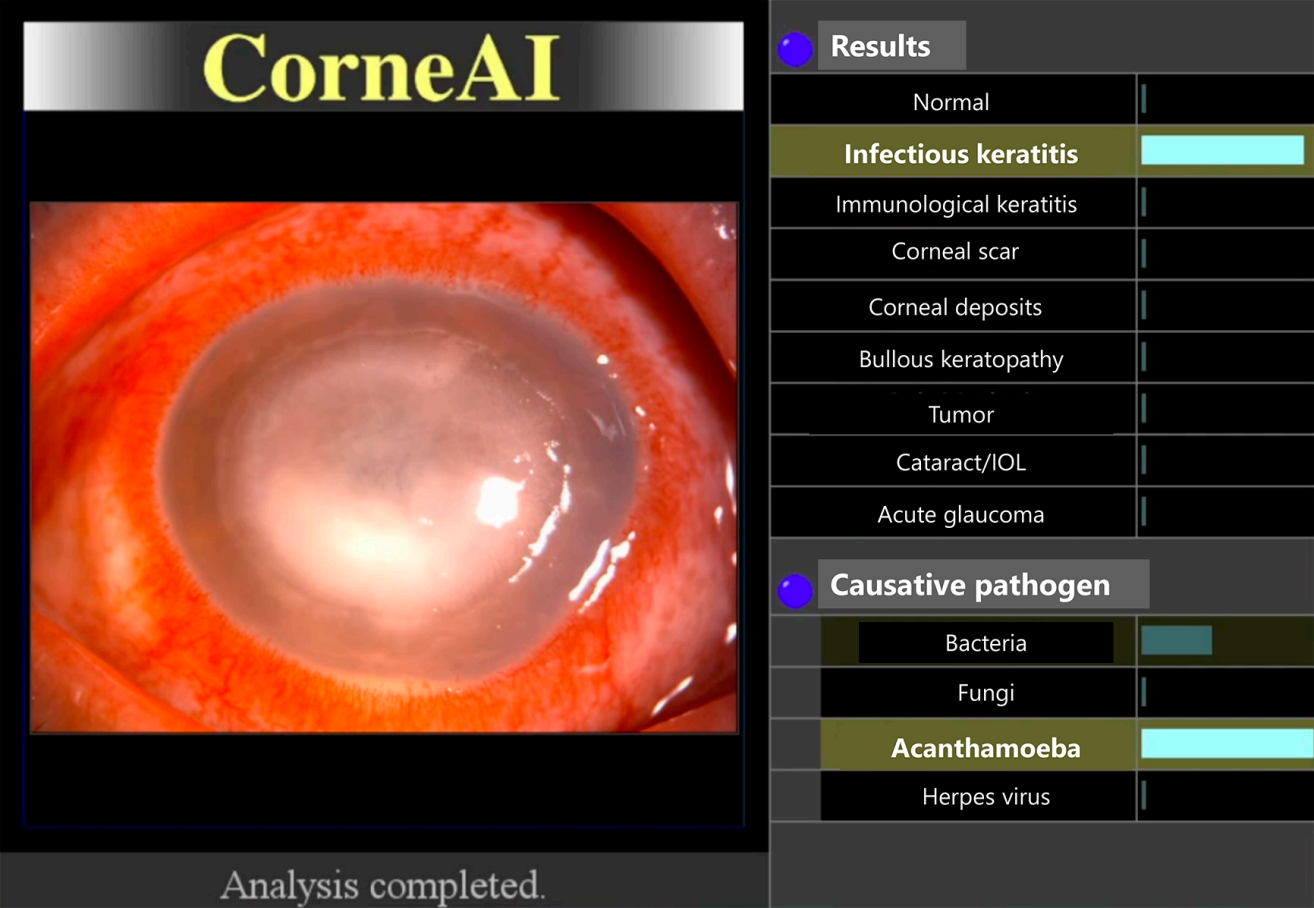
AI application to assist in the differentiation of nine anterior segment conditions. The condition with the highest likelihood is highlighted ^(8)^. In cases of infectious keratitis, the most likely pathogen is highlighted ^(10)^.

Differential diagnosis in corneal diseases is both challenging and critically important owing to the wide range of potential etiologies and the need for prompt and appropriate treatment. One of the most important distinctions that must be made is between infectious and noninfectious corneal diseases, as this determines whether antibiotics or steroids should be used. Infectious keratitis, caused by bacteria, viruses, fungi, or acanthamoeba, requires specific antimicrobial treatments, whereas noninfectious keratitis, which can result from autoimmune diseases, dry eye syndrome, or toxin exposure, is often treated with corticosteroids or other anti-inflammatory medications. Misuse of treatments, such as applying steroids for infections, can lead to adverse outcomes, highlighting the critical need for precise differential diagnosis. The overlap in clinical features, such as redness, pain, photophobia, and decreased vision, along with the rapid progression of corneal diseases, makes timely and accurate diagnosis essential to prevent complications, such as corneal perforation, scarring, and vision loss. Accurate diagnosis often requires specialized tools and expertise, which may be limited in under-resourced settings. Advances in AI and diagnostic technologies hold promise for improving the accuracy and speed of these diagnoses, ultimately enhancing patient care and outcomes in ophthalmology. Several systems have been developed for the differential diagnosis of various anterior segment conditions (Figure 2) ^(7), (8), (9)^. Moreover, deep-learning algorithms applied to slit-lamp images can now estimate the most probable causative pathogen in cases of infectious keratitis ^(10)^.

## 3. Smartphone Imaging

**Figure 3.**
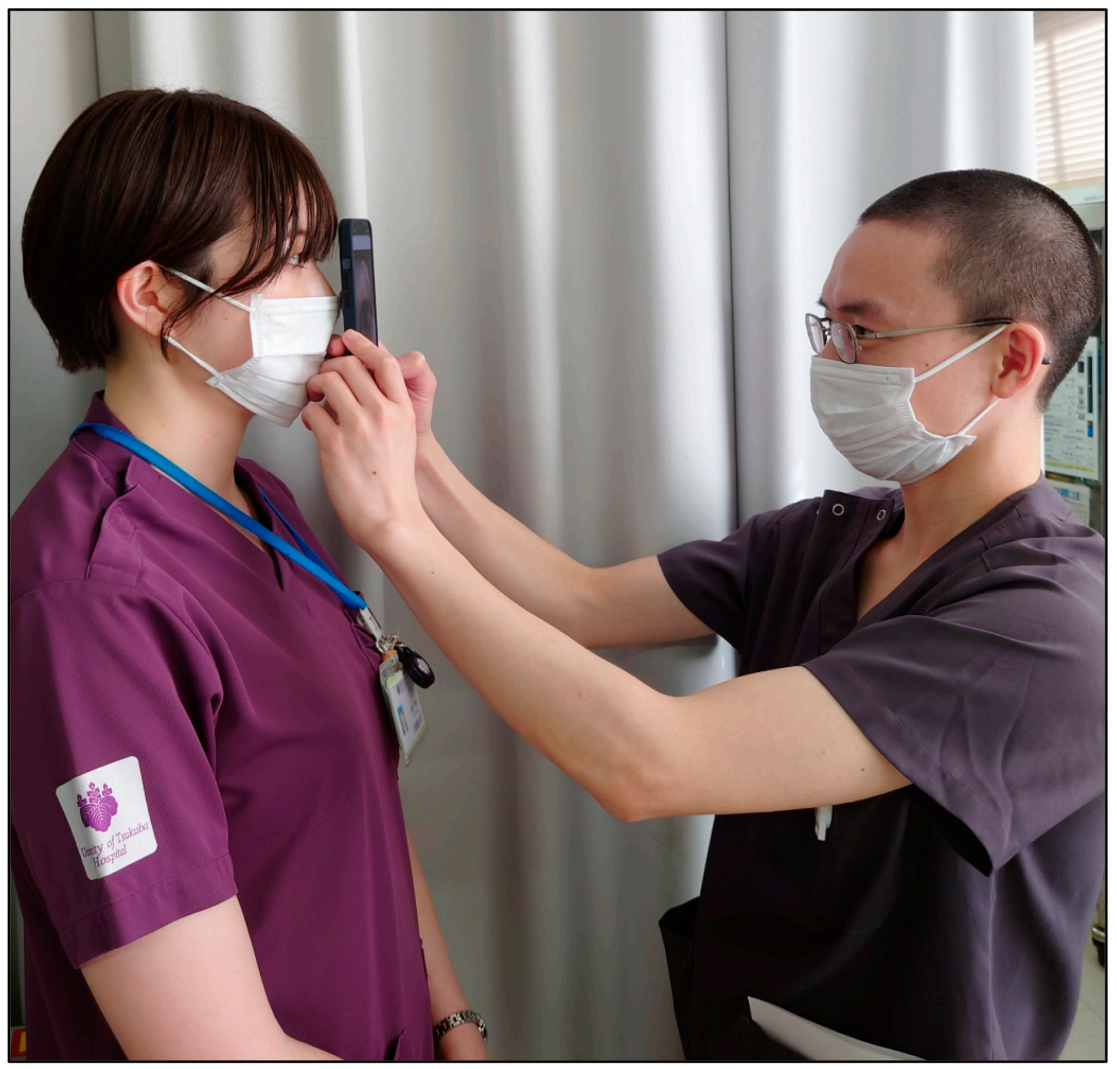
AI application for the differential diagnosis of anterior segment diseases can be applied to photos taken with smartphones ^(8)^.

The use of smartphone imaging coupled with AI technology can significantly advance the diagnosis of anterior segment diseases (Figure 3) ^(8), (11), (12)^. Smartphone cameras, equipped with high-resolution capabilities, are capable of capturing detailed images of the anterior segment of the eye, including the cornea, conjunctiva, and eyelid. When these images are analyzed using AI algorithms, particularly deep-learning models, the system can accurately identify and differentiate various anterior segment conditions, such as keratitis, conjunctivitis, and corneal dystrophies.

**Figure 4.**
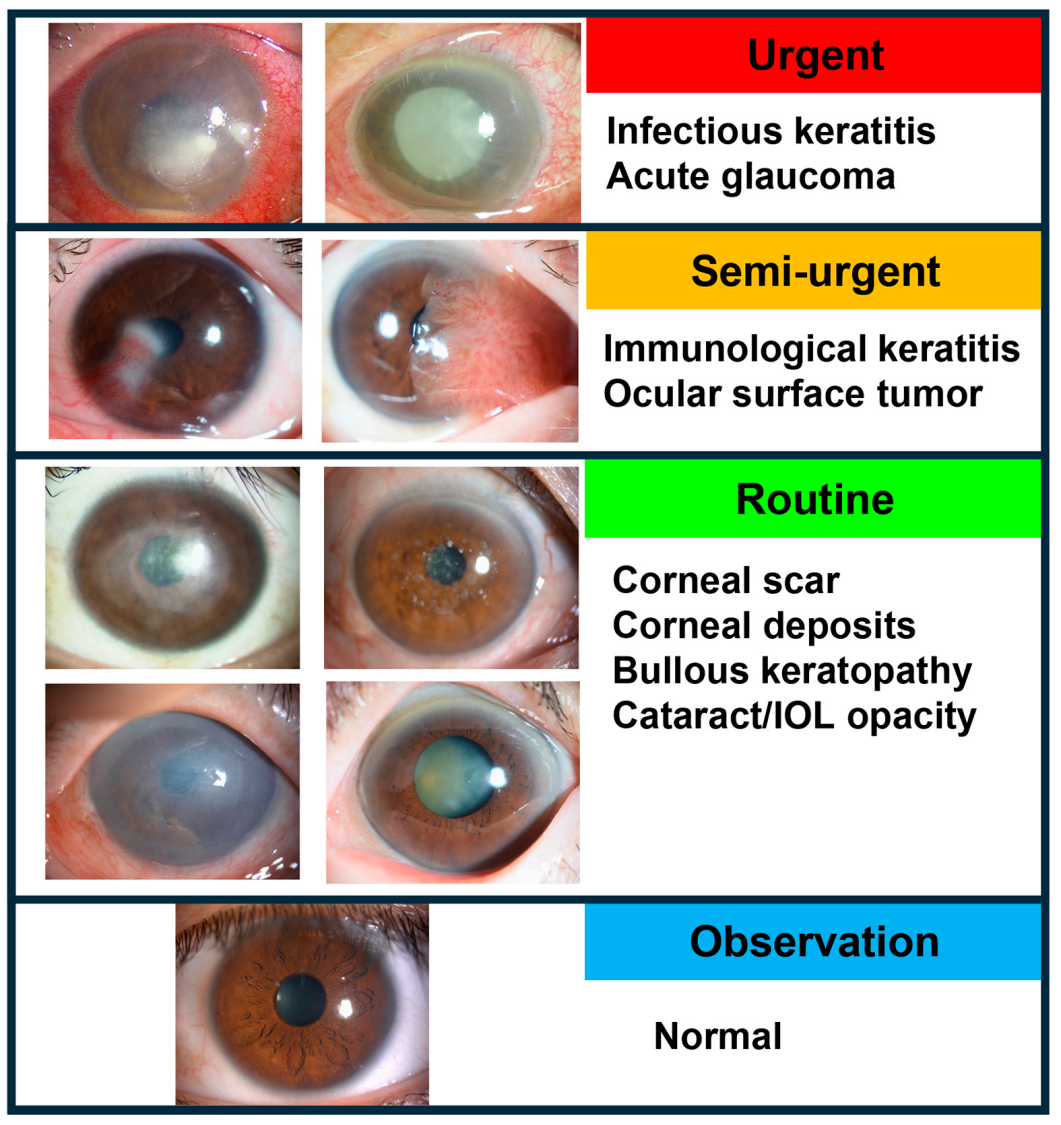
An image of the triage system for anterior segment diseases obtained using smartphone images.

The advantages of this approach are manifold. First, it democratizes access to eye care by allowing patients in remote or underserved areas to receive accurate diagnoses without the need to visit specialized clinics. This is particularly beneficial in regions with shortage of ophthalmologists. Second, AI-powered smartphone imaging provides rapid and reliable diagnostic results, enabling timely intervention and treatment. The system can highlight the condition with the highest likelihood, improving the accuracy of diagnoses and facilitating clinical decision-making (Figure 4). Overall, the integration of smartphone imaging with AI enhances the accessibility, speed, and accuracy of diagnosing anterior segment diseases, ultimately leading to better patient outcomes.

## 4. Application of Artificial Intelligence in Various Diseases

### a. Diabetic retinopathy

The prevalence of diabetes has tripled worldwide over the past two decades, causing a substantial increase in the incidences of microvascular damage and retinal dysfunction due to chronic hyperglycemia. Approximately one-third of individuals with diabetes develop diabetic retinopathy (DR), which is a major cause of vision loss among working-age adults. Early identification and timely treatment of DR can prevent 95% of blindness caused by this condition, prompting the World Health Organization to recommend regular DR screening for patients with diabetes ^(13)^. However, large-scale screening programs require significant manpower and resources, which poses a challenge, particularly in low- and middle-income countries.

The emergence of AI provides innovative solutions to these challenges by enabling automated screening and referral systems for DR ^(14), (15)^. AI algorithms, particularly those based on deep learning, can analyze retinal images with high accuracy, identifying and grading the severity of DR. These systems can quickly and efficiently process large volumes of images, reducing the burden on ophthalmologists and improving screening coverage. The ability of AI to detect early-stage DR and predict disease progression is crucial for timely intervention, which can significantly delay or even reverse the progression of the disease. In addition, AI-driven predictive models can optimize screening intervals and allocate resources more effectively, ultimately lowering costs and improving patient outcomes. A recent review showed that AI algorithms have acceptable performance in screening for DR using fundus images compared with human graders ^(16)^. The implementation of a fundus camera with AI-based software has the potential to assist ophthalmologists in reducing their workload and improving the accuracy of DR diagnosis.

### b. Age-related macular degeneration

Age-related macular degeneration (AMD) is a disease affecting the macular area of the retina; it often leads to progressive loss of central vision. As the population ages, AMD is projected to remain as a major cause of vision impairment worldwide. Timely screening and medical intervention for AMD, particularly neovascular AMD, can reduce vision loss and improve patient outcomes.

AI has significant potential in facilitating the automated detection of AMD and predicting its progression ^(17), (18), (19)^. AI systems, leveraging deep-learning algorithms, can analyze fundus images to detect early signs of AMD, such as large drusen and pigmentary abnormalities, and classify different stages of the disease with high accuracy. In addition, AI can predict the progression to late-stage AMD, guiding high-risk patients in adopting preventive measures and helping clinicians determine appropriate follow-up intervals. By incorporating genetic and environmental factors, AI approaches will further enhance the prediction of AMD progression, enabling more personalized and timely interventions.

### c. Retinopathy of prematurity

Retinopathy of prematurity (ROP) is a proliferative retinopathy affecting premature or low-birth-weight infants. AI has shown significant potential in the automatic diagnosis and staging of ROP ^(20), (21), (22)^. AI models, using deep-learning techniques, can analyze retinal images to accurately diagnose ROP and determine its stage, often outperforming human experts. These systems enhance the efficiency and accuracy of ROP screening, reducing the workload on clinicians and enabling timely interventions. The ability of AI to detect and stage ROP, assess disease severity, and predict disease progression makes it a valuable tool in the clinical management of ROP. By improving diagnostic capabilities and facilitating early treatment, AI contributes to better visual outcomes for infants at risk of this sight-threatening condition.

### d. Vitreoretinal surgery

Robotic surgery offers numerous advantages, particularly in precise movement control and the elimination of tremors, although its application in ophthalmology is still emerging. These systems, enhanced with digital imaging, improve depth perception, which is crucial for delicate tasks, such as subretinal drug delivery and retinal vascular cannulation ^(23), (24)^. Robotic systems can be handheld, teleoperated, co-manipulated, or fully automated, with magnetically controlled systems enhancing intraocular maneuverability, despite the need for a larger surgical site. Reinforcement learning and deep-learning techniques were developed to improve robotic performance in tasks such as intraoperative OCT-guided surgery and subretinal injections ^(25)^. Deep-learning approaches were also developed to estimate the distance between the tool and layers from intraoperative OCT for robotic subretinal injections ^(26), (27)^.

### e. Glaucoma

Glaucoma, characterized by optic disc cupping and visual field impairment, is a leading cause of irreversible blindness, affecting over 70 million people worldwide. Early diagnosis and timely treatment can prevent most vision loss caused by glaucoma. However, early detection of types such as primary open-angle glaucoma, normal tension glaucoma, and chronic primary angle-closure glaucoma is challenging. These forms of glaucoma are often painless, and early visual field defects are subtle, leading to late-stage self-detection when central visual acuity is already compromised. In addition, traditional methods of glaucoma detection, which involve optic disc and retinal nerve fiber layer examination by specialists, are time-consuming and labor-intensive, making them impractical for large-scale screening.

AI offers promising solutions for cost-effective glaucoma screening through automated analysis of fundus and OCT images ^(28), (29), (30)^. AI systems can detect glaucomatous optic neuropathy with high sensitivity and specificity, significantly improving the efficiency of screening programs. Unlike fundus imaging, which captures two-dimensional images of the optic nerve head, OCT provides three-dimensional imaging that can detect depth-resolved structural changes in glaucoma. AI models trained on OCT images have exhibited superiority to those trained on fundus images in terms of accuracy, performing comparably to experienced glaucoma specialists. AI can also predict glaucoma progression by analyzing visual field data, identifying disease progression earlier than traditional methods. These advancements in AI technology enhance early detection and monitoring of glaucoma, facilitating timely interventions and better patient outcomes.

### f. Keratoconus

The diagnosis and management of corneal diseases are highly dependent on imaging modalities, such as slit-lamp photography, corneal topography, anterior segment OCT, specular microscopy, and *in vivo* corneal confocal microscopy. These imaging techniques provide valuable data that can be utilized to train AI algorithms for the automated diagnosis and screening of corneal conditions.

Keratoconus, a progressive corneal ectasia characterized by thinning and protrusion of the cornea, leads to irreversible visual impairment because of irregular corneal astigmatism or loss of corneal transparency. Early identification of keratoconus, particularly in subclinical cases, and timely treatment (e.g., corneal crosslinking and intrastromal corneal ring segments) are crucial to disease stabilization and visual outcome improvement.

AI has shown significant promise in accurately diagnosing both keratoconus and subclinical keratoconus and predicting their progression trends ^(31), (32), (33)^. Using machine learning and deep-learning techniques, AI models can analyze data from various corneal imaging modalities to detect subtle changes indicative of keratoconus that might be missed in conventional examinations. These models can achieve high accuracy, sensitivity, and specificity in identifying keratoconus, providing an effective tool for early detection. Moreover, AI can predict the progression of keratoconus, aiding clinicians in making personalized management plans and optimizing the timing of interventions, such as corneal crosslinking and follow-up visits. This capability enhances the prudent and cost effective use of treatments, ultimately improving patient care and outcomes in the management of keratoconus.

### g. Cataract

Cataract is the leading cause of avoidable blindness and vision loss worldwide. Cataract extraction is the most prevalent surgical procedure of all medical specialties.

AI models can analyze slit-lamp images to detect and quantify various types of cataracts, such as nuclear sclerosis, cortical lens opacity, and posterior subcapsular cataract, with high accuracy ^(34), (35)^. This automated classification can significantly increase the accessibility and efficiency of cataract evaluation globally. In addition, AI systems developed using fundus images for cataract screening have demonstrated high accuracy, offering a single-imaging modality solution that can be integrated into existing AI systems for simultaneous screening of other posterior segment diseases ^(36)^.

Aside from screening, AI also enhances the surgical experience for phacoemulsification cataract surgery ^(37)^. AI-driven platforms can provide real-time guidance during surgery by analyzing video frames, locating the pupil, identifying surgical phases, and offering visual feedback to surgeons. This capability improves precision during surgery and is particularly useful for handling complex cataract cases. Overall, the integration of AI into cataract diagnosis, screening, and surgery offers significant potential to improve patient outcomes and reduce the global burden of cataract-related blindness.

**Figure 5.**
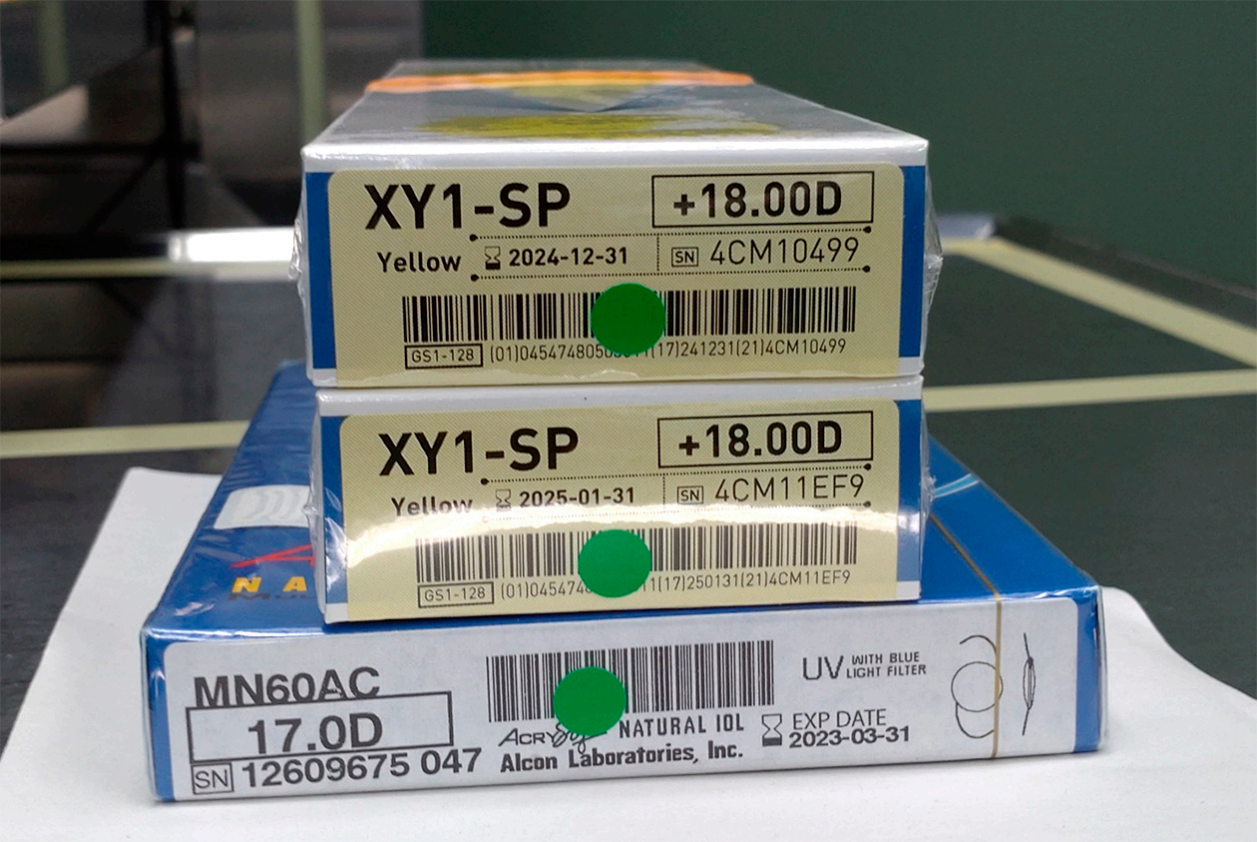
Intraocular lens packages. A mobile device camera is used to capture the image to recognize the parameters shown on the packages of planned, reserved, and backup lenses. The AI system verifies the accuracy of these parameters before surgery ^(38)^.

An AI-based system can be used for preoperative safety management in cataract surgery, focusing on facial recognition, laterality confirmation, and intraocular lens (IOL) parameter verification (Figure 5) ^(38)^. Using deep-learning models, the system achieved high accuracy in facial recognition, laterality confirmation, and IOL parameter verification, with both false rejection and false acceptance rates at 0% after authentication. This AI system effectively enhanced preoperative safety management in cataract surgeries.

### h. Oculoplastics

In oculoplastics, AI offers significant potential for enhancing both pre- and postoperative evaluations and providing decision support for referrals by nonspecialists ^(39)^. AI-powered semantic segmentation networks can automate periorbital measurements with accuracy comparable to that of human graders. Moreover, AI models have been trained to assist general practitioners in making referral decisions for blepharoptosis, demonstrating higher sensitivity and specificity than nonophthalmic physicians’ referrals. In addition, AI algorithms have been created to objectively monitor patient progress after eyelid surgery, ensuring that evaluations are standardized and consistent ^(40)^.

### i. Other anterior segment diseases

The advancement of AI technology has led to significant research into the use of intelligent models for diagnosing pterygium ^(41)^. Although AI-based pterygium diagnosis systems are still in their infancy, the technology shows promise for future clinical applications, including the potential use of smartphone images for diagnosis ^(42)^. AI systems have been developed to detect obstructive and atrophic meibomian gland dysfunction (MGD) using *in vivo* laser confocal microscope images, achieving high accuracy, sensitivity, and specificity ^(43)^. Another AI system was introduced to detect malignant eyelid tumors from photographs taken using ordinary digital cameras, showing high precision and accuracy in external tests ^(44)^. In addition, a deep-learning system, based on anterior segment OCT images, identified dry eye disease with notable accuracy, sensitivity, and specificity. This system outperformed traditional clinical tests such as Schirmer’s test and corneal staining and showed comparable results to tear breakup time and the Ocular Surface Disease Index ^(45)^. These advancements indicate the significant potential of AI in improving the diagnosis and management of anterior segment diseases.

### j. Pediatric ophthalmology

The integration of AI into ophthalmic screening and treatment practices offers substantial potential for optimizing pediatric care. Advanced deep-learning algorithms have been designed to evaluate strabismus from external images, facilitating tele-ophthalmology applications ^(46), (47)^. AI systems for strabismus detection also employ eye-tracking deviations and retinal birefringence scanning ^(48)^. Moreover, machine learning technologies are promising for screening high myopia and other refractive errors and identifying children at risk for reading disabilities. A machine learning system using Brückner pupil red reflex imaging and eccentric photorefraction has been developed to detect amblyogenic features of strabismus and high refractive errors, significantly enhancing early detection and intervention ^(49)^.

## 5. Generative Artificial Intelligence

Deep-learning technology has seen exponential growth in ophthalmology, with generative adversarial network (GAN) emerging as a particularly successful model since its introduction in 2014. GANs involve the concurrent training of two models: a generative model that creates images using training data and a discriminative model that determines whether the images are real or generated ^(50)^. This adversarial relationship improves both the models’ performance until the generative model can produce synthetic images nearly indistinguishable from genuine ones.

Recently, GANs have been applied in ophthalmology for various purposes, including predicting treatment response ^(51)^, enhancing the statistical power of clinical trials ^(52)^, removing shadows in OCT images ^(53)^, generating angiography images from retinal fundus photographs ^(54)^, predicting postoperative appearance ^(55)^, detecting glaucoma ^(56), (57)^ and MGD ^(58)^, and synthesizing OCT images for training datasets ^(59)^.

**Figure 6.**
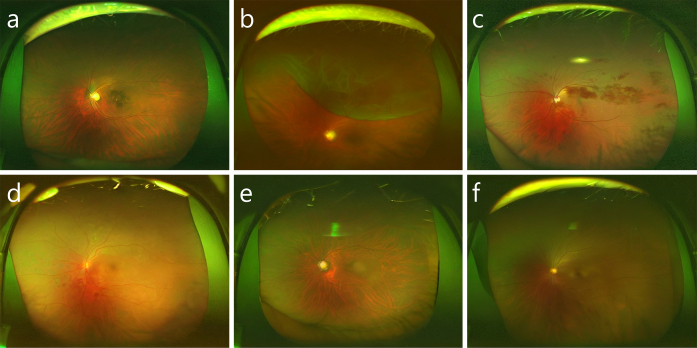
Retinal images were artificially generated using a stable diffusion model. (a) Age-related macular degeneration, (b) retinal detachment, (c) retinal vein occlusion, (d) diabetic retinopathy, (e) glaucoma, and (f) normal fundus.

The use of latent diffusion models, an AI-aided teaching method was developed to train students on numerous images while preserving patient privacy ^(60)^. The training course involved the use of synthetic ultra-widefield retinal images, generated by fine-tuning a large generative model with real images from six categories of retinal diseases and normal conditions (Figure 6). The incorporation of these synthesized images into the training significantly improved students’ diagnostic accuracy. The study demonstrated that synthetic images effectively enhance medical education and highlighted the importance of human judgment in medical diagnostics. This approach could be applied to other medical imaging fields, training experts globally and improving patient outcomes efficiently, showcasing the potential of AI to augment human skills rather than replace them.

## 6. Limitations and Challenges

AI demonstrates significant potential in diagnosing, identifying, screening, staging, and grading various ocular diseases yet faces several challenges that may hinder its further research and clinical application. These challenges include the need for high-quality images, as factors such as equipment type, operator skill, and patient cooperation influence image quality. Accurate manual annotations by experts are crucial, but this process is time-consuming and resource-intensive. Larger sample sizes for datasets are necessary to improve model accuracy, and patient heterogeneity must be considered, as differences in demographics can affect performance. In addition, discrepancies between research settings and real-world clinical environments impact AI performance, and a lack of AI-related knowledge among clinicians leads to the “black-box phenomenon,” where the decision-making process of AI models is not well understood ^(61)^.

Cost effectiveness is another issue. The majority of previous studies concluded that the implementation of AI in ophthalmology is cost effective or cost saving ^(62)^. This suggests that AI has the potential to provide economic benefits in the field. However, the majority of these studies focused on provider perspectives, suggesting that a societal perspective could better inform policymakers. Challenges include ethical considerations such as patient privacy and data security, patient compliance, lack of clear regulations, and high costs of AI implementation ^(63), (64), (65), (66)^. Addressing these issues is crucial for robust health economic assessments. Current research mainly targets major retinal diseases, with further studies needed for conditions such as childhood myopia and cataracts ^(67), (68)^. Regional differences in economic development and medical resources also affect data applicability, highlighting the need for more localized research, particularly in developing countries, to ensure relevant and tailored strategies for AI integration in ophthalmic care. Addressing these issues is crucial for enhancing the effectiveness of AI in ocular disease management and ensuring its successful integration into clinical practice.

## 7. Conclusion

In ophthalmology, AI has the potential to increase patient access to screenings and clinical diagnoses while reducing healthcare costs, particularly in high-risk populations or financially constrained communities ^(69)^. AI is set to transform the field by enhancing diagnostic accuracy, optimizing treatment strategies, and improving patient care. The integration of AI technologies offers unprecedented opportunities for advancement, equipping clinicians with powerful tools to overcome complex challenges. As AI continues to evolve, it will play an increasingly integral role in ophthalmology, driving innovations that enhance patient outcomes and redefine standards of care. The future of ophthalmology is promising, with AI leading this exciting transformation.

## Article Information

### Conflicts of Interest

Alcon, HOYA Medical, Johnson & Johnson Vision, KOWA, Otsuka, Santen, Senju, Tomey Co., Topcon Medical, Futaba, Chugai, Personal financial interest: Logic & Design, Consultant: Alcon, Johnson & Johnson Vision, Santen, HOYA Medical, HOYA Vision Care, Topcon Medical, Logic & Design, Lecture honorarium: Alcon, Glaukos Japan, HOYA Medical, Johnson & Johnson Vision, KOWA, Santen, Senju, Tomey Co., Topcon Medical, Novartis, Inami, Logic & Design, Chugai, Bayer
